# Improved McClelland and Koolen–Moulton bounds for distance energy of a graph

**DOI:** 10.1186/s13660-018-1927-0

**Published:** 2018-12-06

**Authors:** G. Sridhara, M. R. Rajesh Kanna, R. Pradeep Kumar, D. Soner Nandappa

**Affiliations:** 1 Post Graduate Department of Mathematics, Maharani’s Science College for Women, Mysore, India; 2 Department of Mathematics, Sri.D. Devaraja Urs Governement First Grade College, Hunsur, India; 3 Department of Mathematics, Malnad College of Engineering, Hassan, India; 4 Department of Mathematics, University of Mysore, Mysuru, India

**Keywords:** 05C50, 05C69, Distance matrix, Distance spectrum, Bounds for distance energy of graph

## Abstract

Let *G* be a graph with *n* vertices and *m* edges. The term energy of a graph *G* was introduced by I. Gutman in chemistry due to its relevance to the total *π*-electron energy of a carbon compound. An analogous energy $\mathcal{E}_{D}(G)$, called the distance energy, was defined by Indulal et al. (MATCH Commun. Math. Comput. Chem. 60:461–472, 2008) in 2008. McClelland and Koolen–Moulton bounds for distance energy were established subsequently by Ramane et al. (Kragujev. J. Math. 31:59–68, 2008). The lower and upper bounds for $\mathcal{E}_{D}(G)$ obtained in this paper are better than the McClelland and Koolen–Moulton bounds.

## Introduction

Let *G* be a simple undirected graph with *n* vertices and *m* edges. The distance between two vertices $v_{i}$ and $v_{j}$ is denoted by $d_{ij}$ and is defined as the length of the shortest path from $v_{i}$ to $v_{j}$. The distance sum $W(G)= {\sum_{i< j}d_{ij}}$ is called the Wiener index of the graph *G*. For simplicity, we write the Wiener index as *W*. The distance matrix of the graph *G* is defined as $A(G)= A_{D}(G)= [d_{ij}]$. Clearly, $A_{D}(G)$ is a symmetric matrix. Its eigenvalues are called *D*-eigenvalues and are ordered in the form $\mu _{1} \geq\mu_{2} \geq\cdots\geq\mu_{n}$. The largest eigenvalue $\mu_{1}$ is called the distance spectral radius of the graph *G*. We also write its absolute eigenvalues in decreasing order as $\rho_{1} \geq\rho_{2} \geq\cdots\geq\rho_{n}$. Given a graph *G*, the distance energy of *G* is defined by $\mathcal{E}_{D}(G)$ = ${\sum_{i=1}^{n}|\mu_{i}| = \sum_{i=1}^{n}\rho_{i}}$. For any vertex $v_{i}$ in the connected graph *G*, the eccentricity $e(v_{i})$ is the distance between $v_{i}$ and a vertex that is farthest from $v_{i}$ in *G*. The minimum eccentricity among the vertices of *G* is called the radius of graph *G*, and the maximum eccentricity among the vertices is called the diameter of the graph *G*, which are respectively denoted by rad$(G)$ and diam$(G)$.

The distance energy is analogous to the ordinary energy of a graph *G*, which is defined as $\mathcal{E}(G)$ = ${\sum_{i=1}^{n}|\lambda_{i}|}$, where $\lambda_{1} \geq\lambda_{2} \geq\cdots\geq\lambda_{n}$ are ordinary eigenvalues of *G* obtained from its adjacency matrix. The studies on the graph energy can be seen in papers [5, 6]. For a detailed survey on applications to the graph energy, see [2–4, 7]. For the ordinary energy, the best known bounds are the Koolen and Moulton upper bound [9, 10] and the McClelland lower bound [12].

For a connected graph *G*, the Koolen and Moulton upper bound [13] for the distance energy in terms of *W*, *M*, and *n* is

1.1 $$ {\mathcal{E}_{D}(G) \leq \biggl(\frac{2W}{n} \biggr) + \sqrt{(n-1) \biggl(2M - \biggl(\frac{2W}{n} \biggr)^{2} \biggr)}} \quad\mbox{for } 2W \geq n, $$

where ${M= \sum_{i< j}^{n}d_{ij}^{2} }$.

In this paper, we show that the upper bound (1.1) can be modified to a better bound for all classes of graphs with $n^{2} \geq4m$. Further results on upper bounds can also be seen in [11].

The McClelland bounds [13] for the distance energy of a graph, which is true for any connected graph *G*, is

1.2 $$ {\sqrt{2M + n(n-1) \bigl\vert \operatorname{det}(A) \bigr\vert ^{\frac{2}{n}}} \leq\mathcal{E}_{D}(G) \leq \sqrt{2Mn}}. $$

The lower bound obtained in this paper is better than that of McClelland. For more studies on the distance energy, we refer to [1, 8, 14].

We use the following two lemmas, which follow from the properties of distance eigenvalues.

### Lemma 1.1

*Let* *G* *be a graph with* $n \geq3$ *vertices and* *m* *edges*. *Let* $\mu_{1} \geq\mu_{2}\geq\cdots\geq\mu_{n}$ *be* *D*-*eigenvalues of* *G*. *Then*

$${\sum_{i=1}^{n}\mu_{i}= 0} \quad \textit{and}\quad {\sum_{i=1}^{n} \mu_{i}^{2}= 2M}. $$

### Lemma 1.2

*If* $\mu_{1}(G)$ *is the distance spectral radius of the graph* *G*, *then* $\mu_{1}(G) \geq\frac{2W}{n}$.

Note that ${M= \sum_{i< j}^{n}d_{ij}^{2} \geq \sum_{i< j}^{n}d_{ij} = W}$ and ${\sqrt{M}= \sqrt{\sum_{i< j}^{n}d_{ij}^{2} } \leq \sum_{i< j}^{n}d_{ij} = W}$.

## The main results

### Lemma 2.1

*If* $\mu_{n}(G)$ *is the smallest distance eigenvalue of the graph* *G* *and* $\rho_{n}(G)$ *is the smallest absolute distance eigenvalue*, *then*

$${\mu_{n}(G)\leq\sqrt{\frac{2W}{n}}} \quad\textit{and}\quad { \rho_{n}(G) \leq \bigl\vert \operatorname{det}(A) \bigr\vert ^{\frac{1}{n}}}. $$

### Proof

For any nonzero vector *X*,

$${\mu_{n}(G) \leq\min_{X \neq0} \biggl(\frac{X'AX}{X'X} \biggr) \leq\sqrt {\frac{J'AJ}{J'J}} = \sqrt{\frac{2W}{n}}}, $$

where *J* is the unit $n\times1$ matrix $J =[1,1,1,\ldots,1]'$. Now consider $\rho_{1}\rho_{2}\rho_{3} \ldots\rho_{n}= |\operatorname{det}(A)|$. Then $\rho_{n}\rho_{n}\rho_{n}\ldots\rho_{n} \leq\rho_{1}\rho_{2}\rho_{3} \ldots\rho _{n} \leq|\operatorname{det}(A)|$ $\Rightarrow {\rho_{n}(G) \leq |\operatorname{det}(A)|^{\frac{1}{n}}}$. □

### Lemma 2.2

*Let* *G* *be a graph with* $n \geq3$ *vertices and* *m* *edges*. *For the largest and smallest distance eigenvalues* $\mu_{1}$ *and* $\mu_{n}$ *of* *G*, ${\mu_{1} + \mu_{n}\leq2\sqrt{\frac{M(n-2)}{n}}}$.

### Proof

For *D*-eigenvalues $\mu_{1} \geq\mu_{2} \geq\cdots\geq\mu_{n}$ of *G*, it is well known that ${\sum_{i=1}^{n}\mu_{i}= 0}$ and ${\sum_{i=1}^{n}\mu_{i}^{2}= 2M}$. Using the Cauchy–Schwarz inequality for $(\mu_{2},\mu_{3},\ldots,\mu _{n-1})$ and $(\underbrace{1,1,\ldots,1)}_{(n-2)\ times}\ (n\geq3)$ we have

$${ \Biggl(\sum_{i=2}^{n-1}\mu_{i} \Biggr)^{2}} \leq \Biggl(\sum_{i=2}^{n-1}1 \Biggr) \Biggl(\sum_{i=2}^{n-1} \mu_{i}^{2} \Biggr), $$

that is, $(-\mu_{1}-\mu_{n})^{2} \leq(n-2)(2M-\mu_{1}^{2}-\mu_{n}^{2})$.

$$\begin{aligned} \therefore (n-2)2M &\geq(\mu_{1} + \mu_{n})^{2} + (n-2) \bigl(\mu_{1}^{2} + \mu_{n}^{2} \bigr) \\ &= (\mu_{1} + \mu_{n})^{2} + (n-2) \bigl(( \mu_{1} + \mu_{n})^{2} -2\mu_{1} \mu_{n}\bigr) \\ &= (\mu_{1} + \mu_{n})^{2}(n-1) -2(n-2) \mu_{1} \mu_{n}. \end{aligned}$$

However, ${ (\frac{\mu_{1} + \mu_{n}}{2} )^{2} \geq\mu_{1} \mu_{n}}$, which implies that ${-\mu_{1} \mu_{n} \geq- (\frac{\mu_{1} + \mu _{n}}{2} )^{2}}$.

Thus ${(n-2)2M \geq(\mu_{1} + \mu_{n})^{2}(n-1) - 2(n-2)\frac{(\mu_{1} +\mu _{n})^{2}}{4}}$ = $(\mu_{1} +\mu_{n})^{2} \frac{n}{2}$.

Hence $\mu_{1} + \mu_{n} \leq2 \sqrt{\frac{M(n-2)}{n}}$. □

## Upper bound for the distance energy of a graph

### Theorem 3.1

*Let* *G* *be a graph with* $n \geq3$ *vertices and* *m* *edges*. *If* $n^{2} \geq4m$, *then*

3.1 $$ {\mathcal{E}_{D}(G) \leq\frac{2W}{n} +\sqrt{ \frac{2W}{n}} + \sqrt {(n-2) \biggl(2M -\frac{2W}{n} - \frac{4W^{2}}{n^{2}} \biggr)}}. $$

*The equality holds iff* *G* *is* $\frac{n}{2}K_{2}$.

### Proof

Applying the Cauchy–Schwarz inequality for $(|\mu_{2}|,|\mu_{3}|, \ldots, |\mu_{n-1}|)$ and $(\underbrace{1,1, \ldots, 1)}_{(n-2)\ times}$, we have

$$\begin{aligned} & { \Biggl(\sum_{i=2}^{n-1} \vert \mu_{i} \vert \Biggr)^{2}} \leq \Biggl(\sum _{i=2}^{n-1}1 \Biggr) \Biggl(\sum _{i=2}^{n-1} \vert \mu_{i} \vert ^{2} \Biggr), \\ & {\bigl(\mathcal{E}_{D}(G)- \vert \mu_{1} \vert - \vert \mu_{n} \vert \bigr)^{2} \leq(n-2) \bigl(2M - \vert \mu_{1} \vert ^{2} - \vert \mu_{n} \vert ^{2}\bigr)}, \\ &\mathcal{E}_{D}(G) \leq \vert \mu_{1} \vert + \vert \mu_{n} \vert + \sqrt{(n-2) \bigl(2M - \vert \mu_{1} \vert ^{2} - \vert \mu_{n} \vert ^{2}\bigr)}. \end{aligned}$$

Let $|\mu_{1}|= x$ and $|\mu_{n}|=y$.

We maximize the function $f(x,y) = x + y + \sqrt{(n-2)(2M - x^{2} - y^{2})}$. Differentiating $f(x, y)$ with respect to *x* and *y*, we have

$$\begin{aligned} & {f_{x} = 1 - \frac{x(n-2)}{\sqrt{(n-2)(2M-x^{2}-y^{2})}}}, \qquad {f_{y} = 1 - \frac{y(n-2)}{\sqrt{(n-2)(2M-x^{2}-y^{2})}}}, \\ & {f_{xx} = - \frac{\sqrt{(n-2)}(2M - y^{2})}{(2M - x^{2}- y^{2})^{\frac {3}{2}}}}, \qquad{f_{yy} = - \frac{\sqrt{(n-2)}(2M - x^{2})}{(2M - x^{2}- y^{2})^{\frac{3}{2}}}}\quad \mbox{and} \\ &{f_{xy} = - \frac{\sqrt{(n-2)}(xy)}{(2M - x^{2}- y^{2})^{\frac{3}{2}}}}. \end{aligned}$$

For maxima or minima, $f_{x} = 0$ and $f_{y} = 0$, which implies

$$x^{2}(n-1) + y^{2} = 2M \quad\mbox{and}\quad y^{2}(n-1) + x^{2} = 2M. $$

Solving these equations, we obtain that ${x=y =\sqrt{\frac{2M}{n}}}$. At this point the values of $f_{xx}$, $f_{yy}$, $f_{xy}$, and ${\Delta = f_{xx}f_{yy} - (f_{xy})^{2}}$ are

$$\begin{aligned} & {f_{xx} = -\frac{\sqrt{(n-2)}(n-1)}{\sqrt{\frac{2M}{n}}(n-2)^{\frac {3}{2}}}} \leq0,\qquad {f_{yy} = - \frac{\sqrt{(n-2)}(n-1)}{\sqrt{\frac {2M}{n}}(n-2)^{\frac{3}{2}}}}, \\ & {f_{xy} = -\frac{\sqrt{(n-2)}}{\sqrt{\frac{2M}{n}}(n-2)^{\frac {3}{2}}}} \quad\mbox{and}\quad {\Delta= \frac{n(n^{2}+3-3n)}{2M(n-2)^{2}}} \geq0. \end{aligned}$$

Therefore $f(x,y)$ attains its maximum value at ${x=y =\sqrt{\frac {2M}{n}}}$, and this maximum value is $f (\sqrt{\frac{2M}{n}}, \sqrt {\frac{2M}{n}} ) = \sqrt{2Mn}$.

However, $f(x,y)$ decreases in the intervals

$$\sqrt{\frac{2M}{n}} \leq x \leq\sqrt{M}\quad\mbox{and}\quad 0 \leq y \leq \sqrt{ \frac{2W}{n}} \leq\sqrt{\frac{2M}{n}} \leq\sqrt{M}. $$

Since ${n^{2} \geq4m}$, $m \leq W \leq M$, and $\sqrt{M} \leq W$, we have

$$\sqrt{\frac{2m}{n}} \leq\sqrt{\frac{2M}{n}} \leq\frac {2W}{n} \leq \vert \mu_{1} \vert \leq\sqrt{M},\qquad 0 \leq \vert \mu_{n} \vert \leq\sqrt{\frac {2W}{n}} \leq\sqrt{ \frac{2M}{n}} \leq\sqrt{M}. $$

Thus $f (|\mu_{1}|,|\mu_{n}| ) \leq f (\frac{2W}{n}, \sqrt{\frac {2W}{n}} )\leq f (\sqrt{\frac{2M}{n}}, \sqrt{\frac{2M}{n}} )$

$$\Rightarrow\quad {\mathcal{E}_{D}(G) \leq\frac{2W}{n} +\sqrt{ \frac {2W}{n}} + \sqrt{(n-2) \biggl(2m -\frac{2W}{n} - \frac{4W^{2}}{n^{2}} \biggr)} \leq\sqrt{2Mn}}. $$

For the graph ${G \simeq\frac{n}{2}K_{2}\ (n=2m)}$, $\mathcal{E}_{D}(G)= n$. Hence the equality holds. □

Now we show that the above bound is an improvement of the Koolen–Moulton bound. Take ${g(x,y) = x + y + \sqrt{(n-1)(2M - x^{2} - y^{2})}}$. Then, clearly, ${f(x,y) \leq g(x,y)}$ for all $(x, y)$ in the given region of *x* and *y*.

Along ${x = \frac{2W}{n}}$, ${f (\frac{2W}{n},y ) = \frac {2W}{n} + y + \sqrt{(n-2) (2M - \frac{4W^{2}}{n^{2}} - y^{2} )}}$. However, ${f (\frac{2W}{n},y )}$ decreases in the interval ${0 \leq y \leq\sqrt{2M - \frac{4M^{2}}{n^{2}}}}$. Since $n^{2} \geq4m$, we also have $0 \leq y \leq\sqrt{\frac{2W}{n}} \leq\sqrt{2M - \frac{4W^{2}}{n^{2}}}$. Thus $f (\frac{2W}{n},\sqrt{\frac{2W}{n}} ) \leq f (\frac {2W}{n}, 0 )$.

Since $f (\frac{2W}{n},0 ) \leq g (\frac{2W}{n},0 )$ and $g(\frac{2W}{n},0) = \frac{2W}{n} + \sqrt{(n-1) (2M - \frac {4W^{2}}{n^{2}} )}$, it follows that $f (\frac{2W}{n},\sqrt{\frac {2W}{n}} ) \leq g (\frac{2W}{n},0 )$. Hence

$$\frac{2W}{n} +\sqrt{\frac{2W}{n}} + \sqrt{(n-2) \biggl(2M -\frac{2m}{n} - \frac{4W^{2}}{n^{2}} \biggr)} \leq \biggl(\frac{2W}{n} \biggr) + \sqrt{(n-1) \biggl(2M - \biggl(\frac{2W}{n} \biggr)^{2} \biggr)}. $$

## Lower bounds for the distance energy of a graph

### Theorem 4.1

*If* *G* *is a nonsingular graph*, *then* $\mathcal{E}_{D}(G) \geq n |\operatorname{det}A|^{\frac{1}{n}}$. *The equality holds iff* *G* *is* $\frac {n}{2}K_{2}$, *where* $n=2m$.

### Proof

For the eigenvalues $\rho_{1}\geq\rho_{2} \geq\cdots\geq \rho_{n}$ of *G* (or its adjacency distance matrix *A*) it is well known that $|\operatorname{det}(A)| = \rho_{1}\rho_{2}\ldots\rho_{n}$. Since *G* is nonsingular, we have $|\operatorname{det}(A)| \neq0$.

Applying the Cauchy–Schwarz inequality for *n* terms $a_{i}=\sqrt{\rho _{i}}$ and $b_{i} =1$ for all $i=1,2,\ldots,n$, We have

$$\begin{aligned} &{\sum_{i=1}^{n}\sqrt{\rho_{i}} \leq\sqrt{ \Biggl(\sum_{i=1}^{n} \rho_{i} \Biggr)n}}, \\ & {\sqrt{\mathcal{E}_{D}(G)} \geq\frac{\sum_{i=1}^{n}\sqrt{\rho_{i}}}{\sqrt {n}} }. \end{aligned}$$

However, ${\frac{\sqrt{\rho_{1}} + \sqrt{\rho_{2}} + \cdots+ \sqrt{\rho _{n}}}{n} \geq (\sqrt{\rho_{1}\rho_{2}\ldots\rho_{n}} )^{\frac{1}{n}}}$,

$$\begin{aligned} {\sqrt{\mathcal{E}_{D}(G)} \geq\frac{n (\sqrt{\rho_{1}\rho_{2}\ldots\rho _{n}} )^{\frac{1}{n}}}{\sqrt{n}}} \quad \Rightarrow\quad {\mathcal{E}_{D}(G) \geq n \vert \operatorname{det}A \vert ^{\frac{1}{n}}}. \end{aligned}$$

For the graph $G\simeq\frac{n}{2}K_{2}$ with $n=2m$, $|\operatorname{det}(A)| =1$. Hence the equality holds. □

### Theorem 4.2

*Let* *G* *be a graph with* $n > 1$ *vertices and* *m* *edges*, *and let* $2W \geq n$. *Then*

4.1 $$\begin{aligned} {\mathcal{E}_{D}(G) \geq\frac{2W}{n} + \frac{(n-1) \vert \operatorname{det}(A) \vert ^{\frac {1}{(n-1)}}}{ (\frac{2W}{n} )^{\frac{1}{(n-1)}}}}. \end{aligned}$$

*The equality holds if* *G* *is isomorphic to* $K_{n}$ *and* $\frac {n}{2}K_{2}$ *with* $n=2m$.

### Proof

Using the Cauchy–Schwarz inequality for $\sqrt{\rho_{2}},\sqrt {\rho_{3}},\ldots,\sqrt{\rho_{n}}$ and $(\underbrace{1,1,\ldots,1)}_{(n-1)\ times}$, we have

$$\begin{aligned} &{\sum_{i=2}^{n}\sqrt{\rho_{i}} \leq\sqrt{ \Biggl(\sum_{i=2}^{n} \rho_{i} \Biggr) (n-1)}}, \\ &{\sum_{i=2}^{n}\sqrt{\rho_{i}} \leq\sqrt{ \bigl(\mathcal{E}_{D}(G) - \rho _{1} \bigr) (n-1)}}, \\ &{\sqrt{\mathcal{E}_{D}(G) - \rho_{1}} \geq \frac{\sum_{i=2}^{n}\sqrt{\rho _{i}}}{\sqrt{n-1}} }. \end{aligned}$$

However, ${\frac{\sqrt{\rho_{2}} + \sqrt{\rho_{3}} + \cdots+ \sqrt{\rho _{n}}}{n-1} \geq (\sqrt{\rho_{2}\rho_{3}\ldots\rho_{n}} )^{\frac{1}{n-1}}}$, and therefore

$$\begin{aligned} &{\sqrt{\mathcal{E}_{D}(G) - \rho_{1}} \geq \frac{(n-1) (\sqrt{\rho_{2}\rho _{3}\ldots\rho_{n}} )^{\frac{1}{(n-1)}}}{\sqrt{(n-1)}}}, \\ &{\mathcal{E}_{D}(G) \geq\rho_{1} + (n-1) ( \rho_{2}\rho_{3}\ldots\rho_{n} )^{\frac{1}{(n-1)}}} = \rho_{1} + (n-1) \biggl(\frac{ \vert \operatorname{det}(A) \vert }{\rho_{1}} \biggr)^{\frac{1}{(n-1)}}. \end{aligned}$$

Let $\rho_{1} = x$ and ${f(x) = x + (n-1) (\frac{|\operatorname{det}(A)|}{x} )^{\frac{1}{(n-1)}}}$. Then ${f'(x) = 1- \frac{|\operatorname{det}(A)|^{\frac{1}{(n-1)}}}{x^{\frac{n}{(n-1)}}}}$ and $f''(x) = \frac{n|\operatorname{det}(A)|^{\frac{1}{(n-1)}}}{(n-1) x^{\frac{(2n-1)}{(n-1)}}}$.

For maxima or minima, $f'(x) = 0$, which gives the value $x = |\operatorname{det} (A)|^{\frac{1}{n}}$.

At this point, ${f''(x) = \frac{n}{(n-1)}|\operatorname{det}(A)|^{\frac{-1}{n}}} \geq 0$ for all $n > 1$. Thus the function $f(x)$ attains its minimum at $x = |\operatorname{det}(A)|^{\frac{1}{n}}$, and the minimum value is ${f(|\operatorname{det}(A)|^{\frac{1}{n}}) = n |\operatorname{det}(A)|^{\frac{1}{n}}}$. However, ${ \frac {2M}{n} = \frac{\rho_{1}^{2} + \rho_{2}^{2} + \cdots+\rho_{n}^{2}}{n}}\geq\frac {2W}{n} \geq { \frac{\rho_{1}+ \rho_{2} + \cdots+\rho_{n}}{n} \geq(\rho _{1}\rho_{2}\ldots\rho_{n})^{\frac{1}{n}}}$. This implies $|\operatorname{det}(A)|^{\frac{1}{n}}\leq \frac{2W}{n}$. Since $2W \geq n$, we have $\frac{2W}{n} \leq\rho_{1}$.

Therefore, the function is increasing in the interval $|\operatorname{det}A|^{\frac {1}{n}} \leq\frac{2W}{n} \leq\rho_{1}\leq\sqrt{2M}$, and therefore ${f(\rho_{1}) \geq f (\frac{2W}{n} )}$, and

$${\mathcal{E}_{D}(G) \geq\frac{2W}{n} + \frac{(n-1) \vert \operatorname{det}(A) \vert ^{\frac {1}{(n-1)}}}{ (\frac{2W}{n} )^{\frac{1}{(n-1)}}}}. $$

(i) If *G* is isomorphic to $K_{n}$, then $|\operatorname{det}(A)|= n-1$, $\frac {2W}{n} = n-1$, and hence $\mathcal{E}_{D}(G) = 2(n-1)$.

(ii) If *G* isomorphic to $\frac{n}{2}K_{2}$ with $n=2m$, then the eigenvalues are ±1 (each with multiplicity $\frac{n}{2}$), and hence $\mathcal{E}_{D}(G) = n$. □

### Theorem 4.3

*Let* *G* *be a graph with* *m* *edges and* $n\ (>3)$ *vertices*, *and let* $W \geq n$. *Then*

4.2 $$\begin{aligned} {\mathcal{E}_{D}(G) \geq \biggl(\frac{W}{n} \biggr) + \biggl(\frac{2W}{n} \biggr) + \frac{(n-2) \vert \operatorname{det}(A) \vert ^{\frac{1}{(n-2)}}}{ ( (\frac{W}{n} ) (\frac{2W}{n} ) )^{\frac{1}{(n-2)}}}}. \end{aligned}$$

### Proof

For $(n-2)$ entries of eigenvalues $\sqrt{\rho_{2}},\sqrt{\rho _{3}},\ldots,\sqrt{\rho_{n-1}}$ and $(\underbrace{1,1,\ldots,1)}_{(n-2)\ times}$, applying the Cauchy–Schwarz inequality, we have

$${\sum_{i=2}^{n-1}\sqrt{\rho_{i}} \leq\sqrt{ \Biggl(\sum_{i=2}^{n-1}\rho _{i} \Biggr) (n-2)}}, $$

that is,

$${\sum_{i=2}^{n-1}\sqrt{\rho_{i}} \leq\sqrt{ \bigl(\mathcal{E}_{D}(G) - \rho _{1} - \rho_{n} \bigr) (n-2)}}, $$

so that

$${\sqrt{\mathcal{E}_{D}(G) - \rho_{1} - \rho_{n}} \geq\frac{ \sum_{i=2}^{n-1}\sqrt{\rho_{i}}}{\sqrt{n-2}} }. $$

Since the arithmetic mean is greater than or equal to the geometric mean, we get

$$\begin{aligned} &{\sqrt{\mathcal{E}_{D}(G) - \rho_{1} - \rho_{n}} \geq\frac{(n-2) (\sqrt {\rho_{2}\rho_{3}\ldots\rho_{n-1}} )^{\frac{1}{(n-2)}}}{\sqrt{(n-2)}}}, \\ &\mathcal{E}_{D}(G) \geq\rho_{1} + \rho_{n} + (n-2) \biggl(\frac{ \vert \operatorname{det}(A) \vert }{\rho _{1}\rho_{n}} \biggr)^{\frac{1}{(n-2)}}. \end{aligned}$$

The equality holds if *G* is $\frac{n}{2}K_{2}$ with $n=2m$ or $K_{n,n}$.

Put $\rho_{1} = x$ and $\rho_{n} = y$. We minimize the right side of the above function. Let $f(x,y) = x + y + (n-2) (\frac{|\operatorname{det}(A)|}{x y} )^{\frac {1}{(n-2)}}$. Then ${f_{x} = 1 - |\operatorname{det}(A)|^{\frac{1}{n-2}} y (xy)^{\frac {-(n-1)}{n-2}}}$,

$$\begin{aligned} & {f_{y} = 1 - \bigl\vert \operatorname{det}(A) \bigr\vert ^{\frac{1}{n-2}} x (xy)^{\frac{-(n-1)}{n-2}}}, \\ &{f_{xx} = \bigl\vert \operatorname{det}(A) \bigr\vert ^{\frac{1}{n-2}} \biggl(\frac{n-1}{n-2} \biggr) y^{2} (xy)^{\frac{-(2n-3)}{n-2}}}, \\ & {f_{yy} = \bigl\vert \operatorname{det}(A) \bigr\vert ^{\frac{1}{n-2}} \biggl(\frac{n-1}{n-2} \biggr) x^{2} (xy)^{\frac{-(2n-3)}{n-2}}}\quad \mbox{and} \\ &{f_{xy} = \bigl\vert \operatorname{det}(A) \bigr\vert ^{\frac{1}{n-2}} \frac{(xy)^{\frac{-(n-1)}{n-2}}}{n-2}}. \end{aligned}$$

For maxima or minima, $f_{x} =0$ and $f_{y} = 0$, which gives ${x y^{\frac{1}{n-1}} = |\operatorname{det}(A)|^{\frac{1}{n-1}}}$ and $y x^{\frac{1}{n-1}} = |\operatorname{det}(A)|^{\frac{1}{n-1}}$. Solving, we get $x = |\operatorname{det}(A)|^{\frac{1}{n}}$ and ${y = |\operatorname{det}(A)|^{\frac{1}{n}}}$. At this point, the values of $f_{xx}$, $f_{yy}$, $f_{xy}$, and ${\Delta= f_{xx}f_{yy} - (f_{xy})^{2}}$ are ${f_{xx}= f_{yy} = (\frac{n-1}{n-2} )|\operatorname{det}(A)|^{\frac{-1}{n}}}$, ${f_{xy} = \frac{1}{n-2}|\operatorname{det}(A)|^{\frac{-1}{n}}}$, and $\Delta= (\frac{n}{n-2} )|\operatorname{det}(A)|^{\frac{-2}{n}} \geq0$ for all $n \neq2$. The minimum value is $f (|\operatorname{det}(A)|^{\frac{1}{n}}, |\operatorname{det}(A)|^{\frac {1}{n}} ) = n|\operatorname{det}(A)|^{\frac{1}{n}}$. However, $|\operatorname{det}(A)|^{\frac{1}{n} }\leq\frac{2W}{n}\leq\rho_{1} \leq\sqrt {2M}$ and $0 \leq\rho_{n} \leq|\operatorname{det}(A)|^{\frac{1}{n}} \leq\frac{2W}{n} \leq\sqrt{2M}$.

For $W \geq n$, $f(x,y)$ increases in the intervals $|\operatorname{det}(A)|^{\frac{1}{n}} \leq\frac{2W}{n} \leq x \leq\sqrt{2M}$ and $0 \leq y \leq|\operatorname{det}(A)|^{\frac{1}{n}} \leq\frac{W}{n} \leq\frac {2W}{n}\leq\sqrt{2M}$, that is, $f(x,y)$ increases in the intervals $|\operatorname{det}(A)|^{\frac{1}{n}} \leq\frac{2W}{n} \leq\rho_{1} \leq\sqrt{2M}$ and $0 \leq\rho_{n} \leq|\operatorname{det}(A)|^{\frac{1}{n}} \leq\frac{W}{n} \leq \sqrt{2M}$. At $\rho_{n} = \frac{W}{n}$, we have

$$f (\rho_{1},\rho_{n} ) \geq f \biggl(\frac{2W}{n}, \frac{W}{n} \biggr)\geq f \biggl(\frac{2W}{n}, \bigl\vert \operatorname{det}(A) \bigr\vert ^{\frac{1}{n}} \biggr)\geq f \bigl( \bigl\vert \operatorname{det}(A) \bigr\vert ^{\frac{1}{n}}, \bigl\vert \operatorname{det} (A) \bigr\vert ^{\frac{1}{n}} \bigr). $$

Therefore ${\mathcal{E}_{D}(G) \geq (\frac{W}{n} ) + (\frac {2W}{n} ) + \frac{(n-2)|\operatorname{det}(A)|^{\frac{1}{(n-2)}}}{ ( (\frac {W}{n} ) (\frac{2W}{n} ) )^{\frac{1}{(n-2)}}}}$. □

## Brief summary and conclusion

In this paper, we established lower and upper bounds for the distance energy of a graph. Across the globe, attempts are being made by researchers to improve these bounds. The lower and upper bounds obtained in this paper improve the McClelland and Koolen–Moulton bounds for the distance energy of a graph.
